# The hydrophobic C‐terminal sequence of transthyretin affects its catalytic kinetics towards amidated neuropeptide Y

**DOI:** 10.1002/2211-5463.12604

**Published:** 2019-03-04

**Authors:** Sukanya Tangthavewattana, Ladda Leelawatwattana, Porntip Prapunpoj

**Affiliations:** ^1^ Department of Biochemistry Faculty of Science Prince of Songkla University Hat Yai Thailand

**Keywords:** amidated neuropeptide Y, C‐terminal sequence, fluorescence‐based assay, kinetics, proteolytic activity, transthyretin

## Abstract

Transthyretin (TTR) is a transporter for thyroid hormone and retinol binding protein that has recently been reported to have proteolytic activity against certain substrates, including amidated neuropeptide Y (NPY). However, the proteolytic activity of TTR towards NPY is not fully understood. Here, we used fluorescence‐based assays to determine the catalytic kinetics of human TTR towards human amidated NPY. The Michaelis constant (*K*_M_) and catalytic efficiency (*k*_cat_/*K*_M_) of TTR proteolysis were 15.88 ± 0.44 μm and 687 081 ± 35 692 m ^−1^·s^−1^, respectively. In addition, we demonstrated an effect of the C‐terminal sequence of TTR. When the C‐terminal sequence of TTR was made more hydrophobic, the *K*_M_ and *k*_cat_/*K*_M_ changed to 12.87 ± 0.22 μm and 983 755 ± 18 704 m ^−1^·s^−1^, respectively. Our results may be useful for the development of TTR as a therapeutic agent with low risk of the undesirable symptoms that develop from amidated NPY, and for further improvement of the *k*_cat_/*K*_M_ of TTR.

**Abbreviations**
Aβ: amyloid β‐peptide
HP: human plasma
HSA: human serum albumin
*k*_cat_/*K*_M_: catalytic efficiency
*k*_cat_: turnover number
*K*_M_: Michaelis constant
NEP: neprilysin
NPY: neuropeptide Y
RBP: retinol binding protein
TTR: transthyretin
*V*_0_: initial velocity

Neuropeptide Y (NPY) is a 36‐amino‐acid peptide that is distributed in several body systems and has diverse roles in several biological processes 1, 2, 3, 4, 5, 6, 7, 8, 9, 10. The primary structure of NPY is highly conserved particularly among mammalian species, suggesting important physiological roles of the peptide in animals 11, 12. Similar to other neuropeptides, NPY is expressed as a biologically inactive peptide and requires peptidylglycine α‐amidating monooxygenase‐dependent amidation of the C‐terminal end to form an active molecule 13. In addition, the neuroprotective effect of the peptide occurs via the activation of Y receptor 14. Overexpression of NPY phenotype was observed with transthyretin (TTR) knockout 15, 16. Regulation of the neuropeptide maturation by TTR through down‐regulation of peptidylglycine α‐amidating monooxygenase expression was suggested as a mechanism 17. Currently, the use of NPY as a therapeutic agent for particular neurodegenerative diseases has been suggested [18, 19, 20; for review, see 21].

Transthyretin is a homotetrameric protein that is expressed in and secreted from the liver into the blood stream, and from the choroid plexus into the cerebrospinal fluid 22, 23. Its main physiological function is as a transporter for thyroid hormone and retinol binding protein (RBP) 24. Recently, proteolytic activity of TTR was revealed 25, but only a few natural substrates have been identified so far, including amidated NPY 23, 26, 27. The cleavage sites for TTR on amidated NPY are arginine residues 33 and 35 in the C‐terminal region 23. Although the proteolysis has been confirmed as necessary for the neuroprotective effects of TTR 28, the catalytic reaction towards its substrates, particularly NPY, is not fully understood.

In the primary structure of TTR, the particular amino acid residues participating in the formation of the central channel where the binding site for thyroid hormone is located have been highly conserved during the evolution of vertebrates. The predominant changes are in the N‐terminal region of the TTR subunit 29, 30. Our previous studies demonstrated a connection between the alteration in length and hydropathy of the amino acid sequence of the N‐terminal segment of TTR and the binding affinities for thyroid hormone and RBP 31, 32. A few changes of amino acid sequence have also been observed in the C‐terminal sequence of TTR 30, 33, 34, 35 and these were demonstrated to lead to an increase in the binding affinity for RBP 36 and catalytic activity towards apolipoprotein A‐I 37. However, the effect of the changes in the C‐terminal region on the catalytic activity of TTR towards amidated NPY remains unknown.

The study of enzyme kinetics could provide insights into the catalytic mechanism and provide a powerful tool for studying the effect of the structural changes on TTR proteolysis. Therefore, in this study, the kinetic parameters of the proteolytic cleavage of human amidated NPY by human TTR were studied, and these were compared to those of pigC/huTTR (also named TTR‐HPc), a human chimeric TTR in which the C‐terminal sequence of human TTR was changed to that of *Sus scrofa* TTR. Our results demonstrated the effect of the hydrophobic sequence on the catalytic activity of TTR towards amidated NPY.

## Materials and methods

### Purification of TTR from human plasma

An ethical approval request form, patient information sheet, and informed consent form were used following approval by the ethical review committee for research in human subjects, Faculty of Science, Prince of Songkla University, Thailand. Human TTR was isolated and purified from plasma by affinity chromatography followed by preparative discontinuous native‐PAGE 31. The purity and concentration of the protein were determined by SDS/PAGE and the Bradford assay 38, respectively.

### Synthesis and purification of pigC/huTTR

Chimeric TTR, pigC/huTTR, consisting of the residues Gly1 to Ala120 of human TTR and Leu121 to Leu130 of *S. scrofa* TTR, was synthesized by using the heterologous gene expression system of *Pichia pastoris*, and purified by preparative discontinuous native‐PAGE as previously described 36, 37.

### Physicochemical properties of TTR

The relative subunit mass and the electrophoretic mobility under the native condition of TTR were determined by gradient SDS/PAGE and native‐PAGE, respectively 37. The crossed‐reactivity of TTR to specific antibody was analyzed by western blotting 32, using sheep polyclonal antibody against serum human TTR (dilution 1 : 4000; Abcam, Cambridge, UK) and horseradish peroxidase‐linked sheep IgG (dilution 1 : 5000; Calbiochem, San Diego, CA, USA) as primary and secondary antibodies, respectively.

### Hydropathy profiles of TTRs

The hydropathy profiles of human native TTR and pigC/huTTR were generated by Kyte–Doolittle 39 plot analysis, using a scanning window of seven amino acid residues.

### On‐gel analysis of human amidated NPY cleavage by TTR

Aliquots (14 μm) of human amidated NPY (Calbiochem) in 50 mm Tris/HCl, pH 7.4 were incubated alone or in the presence of TTR (2.8 μm) at 37 °C for various times. Thereafter, the reaction mixtures were mixed with a loading buffer containing 1% SDS, and 1% β‐mercaptoethanol, then, without heating, immediately analyzed by Tricine SDS/PAGE (16.5% T, 6% C resolving gel; 4% T, 3% C stacking gel). The proteins were detected by Coomassie Blue G‐250 (Bio‐Rad Laboratories, Hercules, CA, USA) or silver staining (Merck Millipore, Burlington, MA, USA). The reaction mixture of human amidated NPY and albumin purified from human serum (HSA) was included as negative control.

### Labeling amidated NPY with Alexa Fluor 488 and characterization

Human amidated NPY was labeled with Alexa Fluor 488 according to the manufacturer's protocol (Invitrogen, Carlsbad, CA, USA). The labeled product was isolated from free dye by gel filtration chromatography on a Bio‐gel P2 column and quantitatively determined by absorption spectrophotometry at wavelengths 280 and 494 nm, the latter of which is the maximum wavelength of free dye. The degree of labeling was calculated from the absorbance of the conjugate solution at 280 and 494 nm, as described by the manufacturer. The analysis of the labeling peptide was performed by Tricine SDS/PAGE. In brief, an aliquot of the labeled peptide was treated in a buffer containing 1% SDS and 1% β‐mercaptoethanol, without heating, prior to analysis by Tricine SDS/PAGE (16.5% T, 6% C resolving gel; 4% T, 3% C stacking gel). The fluorescence of the labeled peptide was observed under UV light and peptide on the gel was detected by staining with Coomassie Brilliant Blue R‐250. The fluorescence spectrum of the labeled amidated NPY was determined using an RF‐1501 spectrofluorophotometer (Shimadzu, Kyoto, Japan) and then compared to the labeled peptide after the cleavage by TTR.

### Determination of the catalytic kinetics of TTR

The kinetic assay of TTR was performed in 384‐well polystyrol microplatten, μClear® (Greiner Bio One, Kremsmünster, Austria) at 37 °C, using Alexa Fluor 488‐labeled human amidated NPY as substrate. The assay reaction (20 μL) contained purified TTR and the labeled human amidated NPY (1–20 μm) in 50 mm Tris/HCl, pH 7.5. The fluorescence intensity of the proteolytic product was monitored every minute for 30 min, at 37 °C in a Synergy HT plate reader (BioTek, Winooski, VT, USA) filters λ_ex_ = 485 ± 20 nm, λ_em_ = 528 ± 20 nm).

Assay buffer alone and the reaction containing the labeled amidated NPY or TTR alone were included as controls in all of the assays. The fluorescence level of the assays was normalized to the controls, and plotted against time. The initial velocity (*V*_0_) of each reaction was determined from the slope of the curve expressed as a relative fluorescence per minute, and then converted to concentration per minute (μm·min^−1^) using a calibration curve of fluorescence intensity *vs* concentration of the labeled amidated NPY. Then, the *V*_0_ *vs* substrate concentration was plotted and fitted with the Michaelis–Menten equation using non‐linear regression. Maximum velocity (*V*_max_) and Michaelis constant (*K*_M_) values were estimated from a Lineweaver–Burk plot. The turnover number (*k*_cat_) was calculated as the ratio of *V*_max_ to total concentration of TTR used in each reaction. The catalytic efficiency (*K*_cat_/*K*_M_) was obtained from the Lineweaver–Burk plot. Mean and standard error of the kinetic values of human native TTR and pigC/huTTR were calculated from six and three replicates, respectively.

### Statistical analysis

The kinetic data between two groups were assessed by Student's *t*‐test, and *P* < 0.05 was considered to be statistically significant.

## Results

### The hydropathy profiles of TTRs

**Figure 1:**
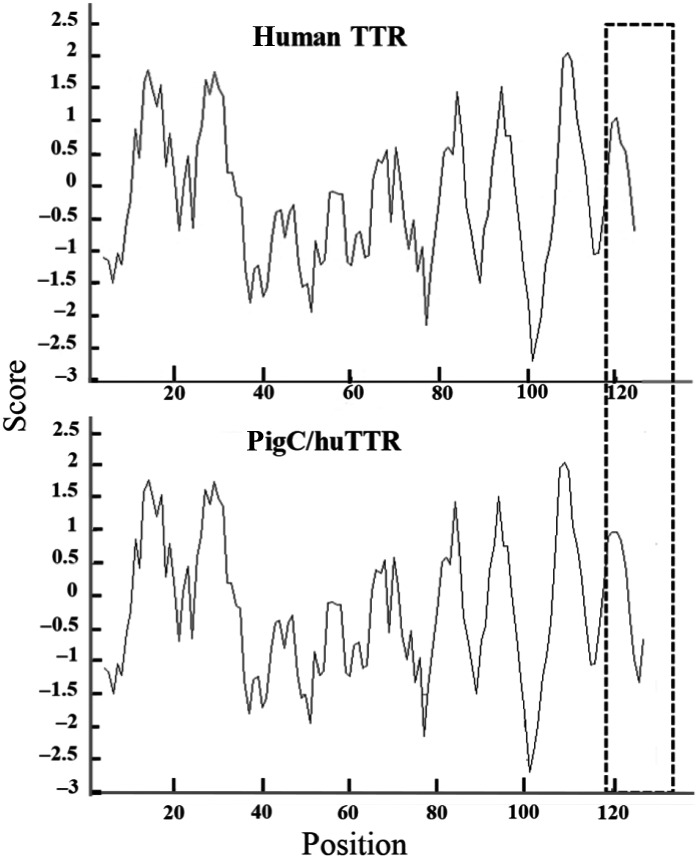
Comparison of hydrophobicity plots of human native TTR and pigC/huTTR. The plots were generated by Kyte–Doolittle plot analysis with window size of seven amino acid residues. The dashed boxes indicate the C‐terminal regions at which the amino acid sequences were changed.

To construct pigC/huTTR, the amino acid residue at positions 121, 123 and 124 of human wild‐type TTR were replaced by leucine, serine and serine, respectively. In addition, three extra amino acid residues, i.e. glycine, alanine and leucine, were added to the C‐terminal end. In the comparison, pigC/huTTR has three more non‐polar amino acid residues than human TTR. Based on the hydropathy profiles of human native TTR and pigC/huTTR generated by Kyte–Doolittle plot analysis, differences in the hydropathy profiles between the two TTRs were observed only at the C‐terminal region (Fig. 1). The C‐terminal sequence of pigC/huTTR showed more hydrophobicity, which was in agreement with a higher number of non‐polar amino acid residues in the C‐terminal region of pigC/huTTR.

### Characterization of purified TTRs

**Figure 2:**
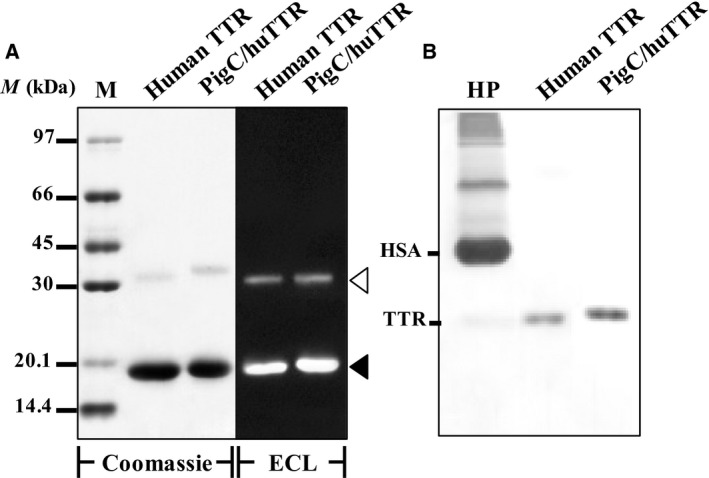
Analysis of TTRs by SDS/PAGE (A) and native‐PAGE (B). (A) Aliquots (2 μg) of purified human native TTR (human TTR) and pigC/huTTR were separated in duplicate by SDS/PAGE (7–15% gradient resolving gel). One of the gels was stained with Coomassie Blue R‐250 (Coomassie) and the other was subjected to western blot analysis using antibody specific to human TTR followed by enhanced chemiluminescence detection (ECL). Standard low molecular mass marker (M) was included. The positions corresponding to monomeric and dimeric forms of TTR are indicated by closed and open arrowheads, respectively. (B) Aliquots (1 μg) of TTRs were separated by native‐PAGE and visualized by Coomassie Blue staining. HP was overloaded to show the positions of TTR and albumin (HSA).

According to SDS/PAGE (7–15% gradient resolving gel, 4% stacking gel) analysis, the subunit masses of human native TTR and pigC/huTTR were 18.0 and 18.7 kDa, respectively. The two had a similar pattern of cross‐reactivity with the specific antibody to human TTR (Fig. 2A). The analysis by native‐PAGE showed that both TTRs were faster than albumin in human plasma (HP) and had a similar mobility to TTR in HP (Fig. 2B).

### On‐gel analysis of the cleavage of human amidated NPY by TTR

**Figure 3:**
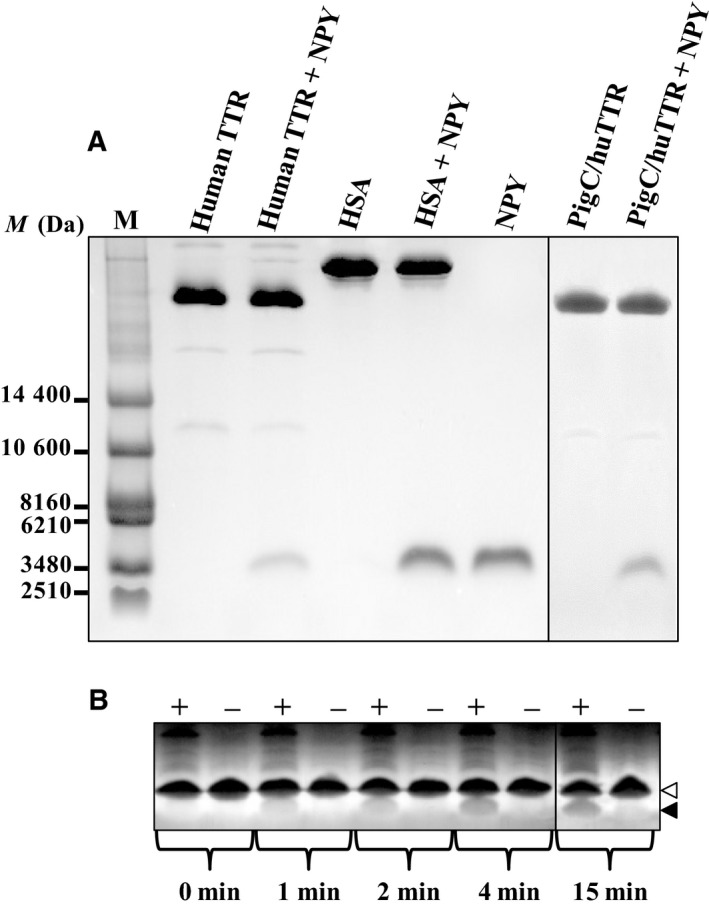
Analysis of NPY cleavage by TTR on Tricine SDS/PAGE. Human amidated NPY (1.5 μg) was incubated alone or with either human native TTR (human TTR) or pigC/huTTR (~ 3 μg) at 37 °C for 1 h prior to analysis by Tricine SDS/PAGE (16.5% T, 6% C resolving gel; 4% T, 3% C stacking gel). For negative control, the amidated NPY was incubated with albumin purified from HSA instead of TTR. The reaction containing TTR or HSA alone was also included. Visualization of the protein bands was carried out by Coomassie Blue staining (A). The cleaved fragment of the amidated NPY with an approximate molecular mass of 2510 Da was observed by silver staining (B). M, standard protein marker.

On Tricine SDS gel, human amidated NPY showed a single band migrating to a position corresponding to a molecular mass of 3480 Da (Fig. 3A). After incubation at 37 °C for 1 h, the intensity of the amidated NPY band in the assay containing either human native TTR or pigC/huTTR significantly decreased compared with that containing amidated NPY or HSA alone. In addition, by silver staining, the cleaved fragment of the amidated NPY with an approximate molecular mass of 2510 Da was clearly observed, particularly within the first 15 min of the incubation with TTR (Fig. 3B).

### Characterization of Alexa 488‐labeled human amidated NPY

**Figure 4:**
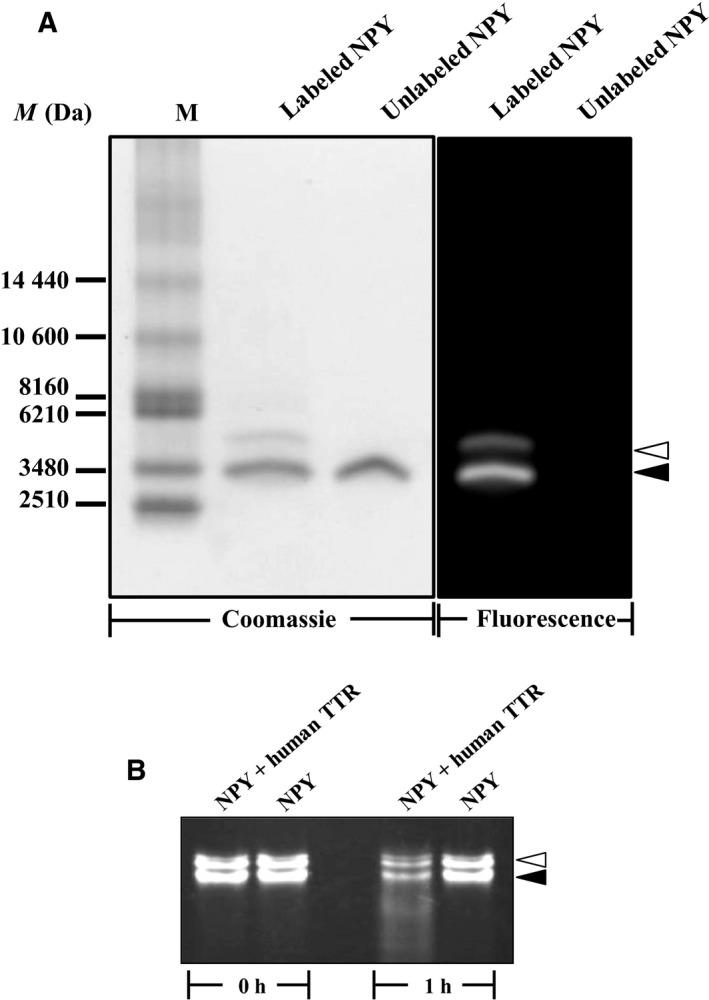
Characterization of fluorogenic human amidated NPY. (A) Aliquots of unlabeled and Alexa Fluor‐labeled human amidated NPY were subjected to analysis by SDS/PAGE. To detect the peptide bands, gel was subjected to Coomassie Blue staining (Coomassie). The fluorescence of the labeled peptide was observed by placing the gel under UV light (Fluorescence). M, standard peptide marker. The positions of lower and upper bands of the labeled NPY are indicated by closed and opened arrowheads, respectively. (B) The labeled human amidated NPY was incubated with or without human native TTR at 37 °C, for 1 h. Then the reaction mixture was analyzed by SDS/PAGE.

Alexa Fluor 488‐labeled human amidated NPY was prepared at pH 8.0 and isolated from free dye by chromatography on a Bio‐gel P2 column. The degree of labeling determined from the absorption spectrum of the labeled peptide was ~ 0.7 mole of dye per mole of NPY. Analysis by Tricine‐SDS/PAGE demonstrated two discrete bands of the labeled amidated NPY, and both of them showed fluorescence under UV light (Fig. 4A). The position on‐gel of the lower band was the same as that of the unlabeled amidated NPY, and its fluorescence intensity was about two‐fold greater than the upper band. These two discrete fluorescence bands were still observed when the labeled amidated NPY was completely denatured by heating in the presence of 8% SDS prior to Tricine‐SDS/PAGE analysis (data not shown). The fluorescence intensity of the two discrete bands decreased with the same rate after incubation with TTR for 1 h (Fig. 4B).

### The catalytic kinetics of TTRs

**Figure 5:**
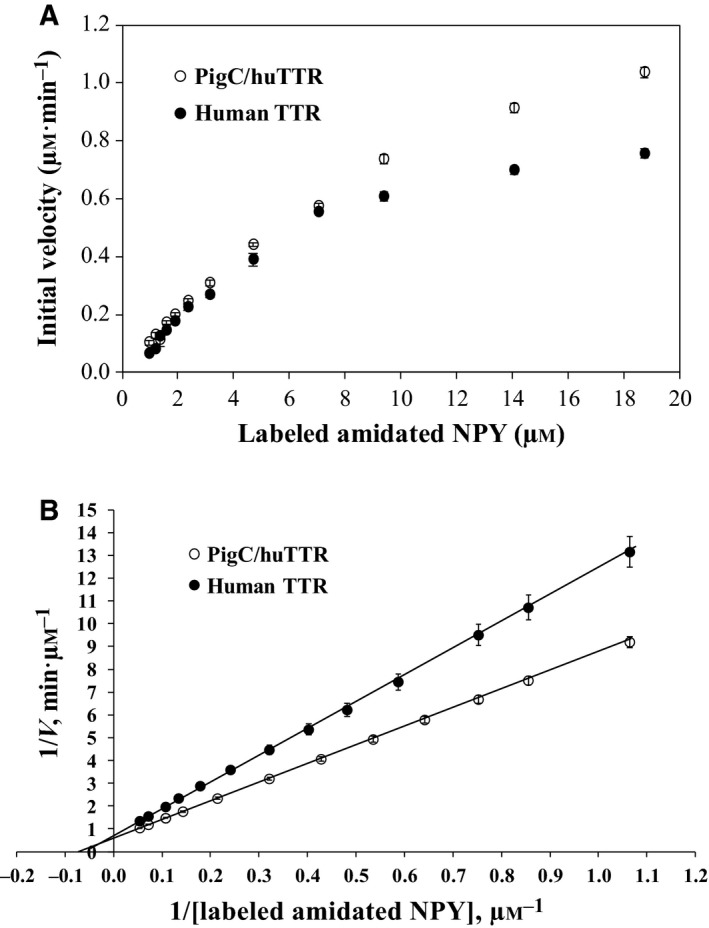
Kinetic plots of the proteolytic reaction of TTR towards human amidated NPY. The proteolytic reactions of the labeled human amidated NPY with human native TTR or pigC/huTTR were performed at 37 °C. The fluorescence intensity of the catalytic reactions was monitored and the *V*_0_ of each reaction was determined. The plot of the *V*_0_ *vs* concentration of the amidated NPY fitted with the Michaelis–Menten equation (A) and a Lineweaver–Burk double reciprocal plot (B) are shown. The kinetic parameters *K*_M_, *k*_ca_ and *k*_cat_/*K*_M_ of the catalytic reactions calculated from the Lineweaver–Burk are presented in Table 1. Error bars indicate the standard error of the mean.

After labeling with Alexa 488, the fluorescence spectrum of the labeled human amidated NPY was not different from that recommended for Alexa Fluor 488 conjugates (i.e. λ_ex_/λ_em_ = 494/519 nm) by the manufacturer. The maxima λ_ex_ and λ_em_ of the labeled amidated NPY were 501 and 521 nm, respectively. By using filters λ_ex_ = 485 ± 20 nm and λ_em_ = 528 ± 20 nm to monitor the fluorescence intensity, it was shown that the fluorescence level of the reaction of the labeled NPY increased immediately the catalytic reaction of TTR was started. The linear increase of the fluorescence intensity of the reaction was observed within the first 10 min or longer depending on the concentration of the labeled amidated NPY. Based on this assay technique, Michaelis–Menten kinetic parameters, including *K*_M_ and *k*_cat_ of human TTR proteolysis towards human amidated NPY, were determined (Fig. 5). It was shown that the affinity for human amidated NPY of human TTR was about 1.2‐fold increased (Table 1) when its C‐terminal sequence was changed from hydrophilic (GRAVY index −0.68) to hydrophobic (GRAVY index 0.26) as observed in pigC/huTTR. In addition, the *K*_cat_/*K*_M_ of pigC/huTTR was significantly ~ 1.4‐fold greater than human TTR (Table 1).

**Table 1:** The kinetic parameters *K*_M_, *k*_cat_ and *k*_cat_/*K*_M_ of the catalytic reactions toward human amidated NPY of human native TTR and pigC/huTTR. The results are presented as means ± standard error of the mean based on *n* replications

Type of TTR	*K*_M_ (μm)	*k*_cat_ (min^−1^)	*k*_cat_/*K*_M_ (m ^−1^·s^−1^)	*n*
Human TTR	15.88 ± 0.44	10.88 ± 0.51	687 081 ± 35 692	6
pigC/huTTR	12.87 ± 0.22	12.65 ± 0.08	983 755 ± 18 704	3

## Discussion

The ability of human TTR to catalyze the cleavage of NPY, the cleavage sites for TTR on the NPY molecule, and the effect of the cleavage by TTR on NPY function were recently revealed 23. In spite of this, relatively few biochemical details about the catalysis of TTR towards NPY are known. Here, fluorescence‐based enzymatic assay was performed to investigate the catalytic kinetics of TTR. In addition, the effect of the C‐terminal sequence on the catalytic activity of TTR was examined.

Post‐translational modifications such as glycosylation and oxidation were reported in human TTR 40, 41, 42. In particular, the modifications of cysteine at position 10 were reported leading to a decrease in the tetrameric stability and, thus, enhanced tetramer dissociation and amyloidogenesis of TTR 43, 44. However, mutation of the residue did not affect the proteolytic activity of TTR 25. On the other hand, glycosylation and its effects on conformational structure and thus function were reported in proteins produced by using the expression system of *P. pastoris* 23, 45. Therefore, we performed an analysis of the relevant physicochemical properties in order to confirm the characteristics of pigC/huTTR and compare them with the human native TTR purified from plasma. Although the apparent subunit masses of the two studied proteins were slightly different from that previously reported for human TTR 34, 36, 41, which was based on the differences of the polyacrylamide percentage of the resolving gel and the efficiency of the analysis method, both proteins showed similar subunit masses and masses of the dimeric forms (~ 31 kDa), and these were in the range of that previously reported for human TTR 34, 36. In addition, the cross‐reactivity with the antibody specific to human TTR and the mobility on‐gel under the native condition of the two proteins were similar to each other, and also to that previously reported for human TTR 36. This confirmed that the human native TTR purified from plasma and the recombinant pigC/huTTR produced by the heterologous gene expression system of *P. pastoris* prepared for the present study had major characteristics similar to native human TTR, including the presence in a tetrameric form and having the proper molecular folding that was necessary for the proteolytic function of TTR.

Neuropeptide Y is widely distributed and its functions are related to several physiological and pathophysiological processes of the body; in addition, the degradation of different parts of the NPY molecule leads to distinct responses in the body 46, 47, 48 (for review, see 49). The analysis of catalysis on Tricine‐SDS gel demonstrated the cleavage of amidated NPY only in the reaction with TTR, not HSA, and the cleavage pattern of the amidated NPY was similar in the catalytic reaction of human native TTR and pigC/huTTR. This confirmed the specificity of the catalytic reaction of TTR towards amidated NPY; however, it also possibly implied a small effect of the C‐terminal sequence on the catalytic activity of TTR. Although the gel‐based assay could provide the conformational state of the catalytic product, there was no clear insight into the interaction between TTR and its substrate in the catalytic reaction. Therefore, the catalytic kinetics of TTR were further determined by a fluorescence‐based assay, and the results significantly demonstrated the effect of the hydrophobicity of the C‐terminal sequence on the catalytic activity of TTR towards NPY in the amidated form.

Among synthetic fluorophores, Alexa Fluor 488 has been successfully used in several labeling techniques 50. In the labeling process, the Alexa dye reacts with primary amines on protein molecules 51, 52, and its conjugate product was claimed to have more fluorescence and be more photostable than the conjugates of conventional dyes 51. Similarly to other dyes in the Alexa series, Alexa 488 is synthesized in succinimidyl ester form, which allows the reaction to take place efficiently at pH 7.5–8.5 53. To label human amidated NPY with Alexa Fluor 488, we followed the standard protocol suggested by the manufacturer at pH 8.0. According to the results of the analysis by SDS/PAGE, the Alexa dye was successfully linked to the amidated NPY.

Within the 36 amino acid residues of human amidated NPY, tyrosine at position 1 and lysine at position 4 contain primary amines as side chains, and these are the targets for Alexa 488. In comparison of the positions of the residues in the molecule of NPY and the p*K*_a_ values of the primary amine (i.e. 9.2 for tyrosine and ~ 10.5 for of lysine), tyrosine at position 1 and lysine at position 4 have a similar potential to interact with Alexa 488 at pH 8.0. Therefore, the labeled amidated NPY could be expected to be in two forms, i.e. with one and two molecules of the Alexa dye attached. These are correlated with the observation of two discrete rather than a single fluorescence band of the labeled amidated NPY and the same rate of decrease in fluorescence level of the two bands upon cleavage by TTR.

The phenomenon of fluorescence quenching of a fluorophore by intrinsic amino acid residues in the protein to which it is linked is known. A few amino acids were demonstrated as fluorescence quenchers for Alexa 488, and tyrosine is one of the strong quenchers 54. In the molecule of human amidated NPY, tyrosine residues were found at positions 20, 21, 27 and 36 55. Only the tyrosine at position 36 is located after the known cleavage sites for TTR. In this study, the fluorescence of Alexa Fluor 488‐labeled amidated NPY increased when the cleavage of the peptide by TTR started. Dynamic quenching, which arises from photo‐induced electron transfer between Alexa Fluor 488 and the phenolic OH group of the tyrosine residues in the amidated NPY 54, possibly occurred, and as a result low fluorescence amidated NPY formed. The cleavage by TTR could lead to loss of tyrosine quencher particularly at position 36, resulting in energy release and, thus, fluorescence intensity detection. In our experiment, the increase of fluorescence was correlated to the cleavage of the labeled amidated NPY; in addition, the changes of the fluorescence increase could be monitored and used for proteolytic kinetics determination. As a result, the proteolysis kinetic parameters, including *K*_M_, *K*_cat_ and *k*_cat_/*K*_M_, of human TTR towards human amidated NPY were first revealed.

Among the NPY‐specific cleaving enzymes that have been identified to date, neprilysin (NEP) and TTR contain specific catalytic reactions toward both NPY and amyloid β‐peptide (Aβ) 23, 25, 26, 48, 56, 57, 58. The NPY peptide fragment generated from the proteolysis of NEP could protect neurons from the toxic effects of Aβ 58. Therefore, exogenous NPY has been suggested as a therapeutic strategy to stimulate the proliferation of progenitor and production of newly generated neurons for several neurodegenerative diseases including Alzheimer's and Parkinson's diseases (for review, sees 21). Despite its role as an angiogenic factor, a higher level of NPY has been reported to be associated with particular pathogenic symptoms and disorders of the central nervous system including anxiety and depression 59, 60. In addition, repeated administration or an abnormally elevated level of NPY was associated with the development of atherosclerotic cardiovascular disease 61 (for reviews, see 62, 63). As a consequence, a therapeutic strategy to prevent NPY‐associated arrhythmias in myocardial infarction and chronic heart failure was suggested (for reviews, see 63, 64). On the contrary, the NPY fragments generated from the cleavage by TTR contain residues 1–33 and 1–35 that lack the ability to bind with their own receptors 23. Therefore, by comparison with Aβ‐degrading enzymes such as NEP, TTR is more beneficial with regard to its specific actions on both Aβ degradation and NPY deactivation, with the latter reducing the pathogenic risk that can arise from the cleaved fragments of NPY.

In the comparison to human TTR, *S. scrofa* TTR has a three amino acid residue extension at the C‐terminal end 33. The hydropathy profile generated by the Kyte–Doolittle method (Fig. 1) and the GRAVY value confirmed more hydrophobicity of the C‐terminal region of pigC/huTTR compared to human TTR. Our previous results demonstrated a relationship between changes of the C‐terminal sequence of TTR subunits and the changes of binding affinity to RBP and catalytic activity towards apolipoprotein A‐I of the protein 36, 37. Here, according to the proteolysis kinetic parameters, i.e. *K*_M_ and *k*_cat_/*K*_M_, which changed from 15.88 ± 0.44 μm in human TTR to 12.87 ± 0.22 μm in pigC/huTTR and from 687 081 ± 35 692 in human TTR to 983 755 ± 18 704 m ^−1^·s^−1^ in pigC/huTTR, respectively, we demonstrated that changing the C‐terminal region of human TTR to be more hydrophobic significantly increased the affinity for human amidated NPY and the *K*_cat_/*K*_M_ of human TTR. In aqueous solution, NPY has a potential to form an amphiphilic secondary structure containing two helical termini, i.e. a left‐handed polyproline II helix in the N‐terminal region (residues 1–13) and an α‐helix in the C‐terminal region (residues 19–32), and the most hydrophobic region of the peptide is between residues 15 and 31. The C‐terminal part of NPY is important for binding to receptors and providing the biological effects, while the N‐terminal part is required for the conformational stability of the whole molecule (for review, see 65). In addition, the hydrophobic interactions with the N‐terminal amphiphilic region stabilized the conformation of the amphiphilic α‐helix (in residues 13–32) and, thus, determined the binding of NPY to cell surfaces 66. An increase of the stability of the polyproline II conformation of the N‐terminal segment, and thus the whole molecule, of the amidated NPY by the hydrophobic environment generated from the hydrophobic C‐terminal sequence is a possible explanation for the decrease of *K*_M_ of the catalytic reaction of TTR towards amidated NPY when its C terminus was changed to a more hydrophobic sequence as observed in *S. scrofa*.

In conclusion, our study is the first to reveal the kinetic parameters of the proteolytic activity of human TTR towards human amidated NPY; in addition, the effect of the hydrophobic C‐terminal sequence on the catalytic activity of the TTR was demonstrated. The present results, together with the reports of the proteolytic activity of TTR towards Aβ, support the beneficial potency of TTR as a therapeutic agent with low risk of the undesirable symptoms that develop from amidated NPY, and in addition, provide information for further improvement of the *K*_cat_/*K*_M_ of TTR.

## Conflict of interests

The authors declare no conflict of interest.

## Author contributions

PP conceived and supervised the study; PP, VP and LL designed the project; ST performed experiments; ST, LL and PP analyzed data; ST wrote the manuscript; and PP revised the manuscript.
